# Child Behavioural Problems and Body Size among 2-6 Year Old Children Predisposed to Overweight. Results From the “Healthy Start” Study

**DOI:** 10.1371/journal.pone.0078974

**Published:** 2013-11-08

**Authors:** Nanna J. Olsen, Jeanett Pedersen, Mina N. Händel, Maria Stougaard, Erik L. Mortensen, Berit L. Heitmann

**Affiliations:** 1 Research Unit for Dietary Studies, Institute of Preventive Medicine, Bispebjerg and Frederiksberg Hospitals, The Capital Region, Copenhagen, Denmark; 2 University of Copenhagen, Copenhagen, Denmark; 3 Department of Public Health and Center for Healthy Aging, University of Copenhagen, Copenhagen, Denmark; 4 The Boden Institute of Obesity, Nutrition, Exercise & Eating Disorders, Sydney Medical School, Sydney University, Sydney, Australia; 5 National Institute of Public Health, University of Southern Denmark, Copenhagen, Denmark; Old Dominion University, United States of America

## Abstract

**Objective:**

Psychological adversities among young children may be associated with childhood overweight and obesity. We examined if an increased level of child behavioural problems was associated with body size among a selected group of 2-6 year old children, who were all predisposed to develop overweight.

**Methods:**

Cross-sectional analyses were conducted using baseline data from the “Healthy Start” intervention study. A total of 3058 children were invited to participate, and data from 583 children who were all predisposed for obesity was analyzed. The Danish version of the Strengths and Difficulties Questionnaire (SDQ) was used to assess child stress by the SDQ Total Difficulties (SDQ-TD) score and the Prosocial Behavior (PSB) score. Height and weight were measured, and BMI z-scores were calculated.

**Results:**

A direct, but non-significant linear trend was found between SDQ-TD score and BMI z-score (β = 0.021, p = 0.11). Having an SDQ-TD score above the 90^th^ percentile was associated with BMI z-score (β = 0.36, p = 0.05). PSB score was not associated with BMI z-score. Analyses were adjusted for parental socioeconomic status, parental BMI, family structure, dietary factors, physical activity, and family stress level.

**Conclusion:**

The results suggested a threshold effect between SDQ-TD score and BMI z-score, where BMI z-score was associated with childhood behavioural problems only for those with the highest scores of SDQ-TD. No significant association between PSB score and BMI z-score was found.

## Introduction

Previous research suggests that the determinants behind the present increase in the obesity prevalence are present already in early childhood, and that obesity is under both genetic and environmental influence [1,2]. Nonetheless, the secular trends in childhood overweight and obesity are not fully explained by changes in traditional lifestyle factors, such as increased food intake and decreased physical activity [3]. In this regard, it has been suggested that psychological adversities also may be a cause of overweight and obesity among children. However, this relationship may be bidirectional [4] since obesity may also predict behavioural problems, as suggested by Sawyer et al. in 2011 [5]. 

Most studies have examined psychological problems in relation to overweight and obesity in school-aged children [6–8], leaving unclear findings for children under the age of 5 [9]. In this regard, a study from 2010 did not find associations between behavioural problems in toddlers and Body Mass Index (BMI) at either age 18 or 36 months, and found no indication that behavioural problems were associated with increasing BMI over time, or vice versa. Based on the negative findings, and the associations between behavioural problems and overweight observed in children above the age of 5, it was concluded that future studies should focus on the age span between 3 and 6 years which is the period that includes the adiposity rebound [9]. Finally, behavioural problems have been found to be more common in children coming from families with low socioeconomic status (SES) [4] which also has been found to be a factor predisposing to development of overweight and obesity [10].

The purpose of the present study was to examine if an increased level of behavioural problems was associated with body size among a selected group of 2-6 year old children, who were all predisposed to develop overweight.

## Materials and Methods

Cross-sectional data was derived from the baseline examination in the intervention study “Healthy Start” [“Sund Start”], which was conducted between 2009 and 2011. The intervention aimed at preventing overweight and obesity in children aged 2-6 years, who were predisposed to overweight because of having either a high birth weight (> 4,000 grams), a mother who was overweight prior to pregnancy (BMI > 28), or a mother with low SES (educational level ≤ 10 years). Information on birth weight and pre-pregnancy BMI of the mother was obtained from the Danish National Birth Register, on all children born between 2004 and 2007 from selected municipalities in the greater Copenhagen area. Information on maternal educational level was obtained from administrative birth forms, filled out by hospital personnel at the time of birth. 

All children eligible to inclusion were randomized into an intervention group, a control group, and a shadow control group. A total of 3,058 children were eligible for inclusion in the intervention and control groups and were invited to attend a first meeting. A total of 635 from the intervention and control groups agreed to participate, and were examined at baseline. In the present study, baseline data from 583 children in the intervention and control groups with information on height and weight as well as level of behavioural problems was used. 

The Healthy Start study has been described in detail previously [11]. 

BMI z-scores were used as measure of relative body fatness. Body height to the nearest 0.1 cm was measured using a stature meter (Soehnle 5002 or Charter ch200P). Body weight to the nearest 0.1 kg was measured using a mechanical weight or beam-scale type weight (Tanita BWB-800 or SV-SECA 710). 

Parents of participating children filled out a questionnaire about life style factors such as family meal habits, the child’s physical activity habits, sleep habits and stress in the family. Unless stated otherwise, all variables described below were obtained from this questionnaire. 

A Danish single-sided version of the Strengths and Difficulties Questionnaire (SDQ) was an integrated part of the parental questionnaire. This rating scale was completed by parents at baseline. The SDQ is a brief questionnaire screening for behavioural problems for 3–16 year old children and adolescents. The SDQ asks about 25 attributes, some positive and others negative. These 25 items are divided into 5 scales (“Emotional symptoms”, “Conduct problems”, “Hyperactivity/inattention”, “Peer relationship problems”, and “Prosocial behaviour”). The scores within all categories except “Prosocial behaviour” were summed to a Total Difficulties score (SDQ-TD score), based on a scoring syntax available from the SDQ-webpage [12], and used as an exposure variable. The score on the Prosocial Behaviour scale (PSB score) was not incorporated into the SDQ-TD score, as absence of prosocial behaviours differs conceptually from the presence of psychological difficulties [13]. The PSB score was hence used as an exposure variable by itself. The SDQ has been completed for nearly 100.000 children and adolescents in both population studies and clinical samples in the Scandinavian countries, and has recently been found to be a useful screening tool across genders and age groups in the general Danish population [14,15]. 

The Parenting Stress Index (PSI) is a self-report inventory designed to measure parental experiences of stress in the parent-child relation [16]. In order to assess the parental perceptions of the family’s well-being in terms of overall stress, 10 questions were selected from the Swedish version and modified according to context. The questions asked which changes in life the parents had perceived since they had had the child regarding 10 dimensions: sleep, stress, worries, time for themselves, household conflicts, work load, social gatherings in the home, joy of life, everyday surplus energy, and complexity of being a parent compared to expectations. The response options to the questions were “more”, “less”, or “no difference” compared to before having the child (e.g. “more sleep”, “less sleep”, or “no difference in sleep”). Each question was then scored between 0 and 2 (with 0 being the best score and 2 being the worst), according to its estimated indication of an overall stress level. Analysis of intercorrelations among the 10 questions and a principal component analysis suggested that 9 of the 10 questions could be added together to get a score for the overall family stress level. This score was further recoded into tertiles. The 10 questions (translated into English from Danish) can be found in Table S1.

Parental height and weight was self-reported in the questionnaire, and based on this information BMI was calculated.

Parental highest level of completed education was reported in 9 categories. Of these categories, 8 were recoded into 3 levels; Low educational level (“primary and lower secondary school”, “upper secondary”, “one or more short courses (ex. semi-skilled worker courses)”, or “skilled worker”), Medium educational level (“short-term further post-high school education (< 3 years)”, “medium-term further education (3-4 years)”), and High educational level (“long-term further education (> 4 years)”, “research worker level”). Responses in the 9^th^ category were not recoded and consequently left out (n = 10 for mothers, and n = 5 for fathers), as they included other education which was not possible to classify according to the originally reported categories (e.g. education completed in foreign countries)

. 

Information on the number of siblings living with the child was obtained, and coded into four categories; “0 siblings living with the child”, “1 sibling living with the child”, “2 siblings living with the child” and “3 or more siblings living with the child”.

Information on household income and on whether or not the parents were divorced was also obtained. Information on parental perception of the child’s physical activity level compared to peers was obtained and used as a proxy for physical activity. The possible answers were “Not as active”, “Fairly active”, “Very active”, and “Do not know”.

Information on dietary intake was obtained from a 4-day dietary record (Wednesday-Saturday) filled out by parents, applying a picture book as guidance in reporting portion sizes.

### Ethics

The Scientific Ethical Committee of the Capital Region in Denmark decided that according to Section 2.-(1) of the Danish Act on a Bioethics Committee System and the Processing of Bioethics Projects, the project was defined not to be a bioethics project and as a result did not need approval from the Danish Bioethics Committee.

Written informed consent to use the collected data for research purpose was obtained from all participants’ parents.

### Statistical methods

BMI z-scores were generated using the Lambda-Mu-Sigma (LMS) method, which summarises the changing distributions of the dependent variable (e.g. BMI) by the median, the coefficient of variation and skew expressed as Box-Cox power [17]. Using z-scores enables comparison of a measured BMI with adequate gender- and age-specific reference values [18]. It was chosen to apply national reference BMI z-score to the study population, and thus, a power transformation of 0.1 years of age was used [18]. As BMI z-score already takes age and gender into account, we did not further adjust the analyses for these factors.

The SDQ-TD score was analysed as a continuous variable, and as a categorical variable after coding the sample into three categories based in internal data, using the 80^th^ and 90^th^ percentile as cut-offs, as recommended by Goodman [13]. Children in the 90^th^ percentile were classified as having an “abnormal score”, children in the 80^th^ percentile were classified as having a “borderline score”, and children below the 80^th^ percentile were classified as having a “normal score” [13]. 

Due to a relatively narrow score range, the PSB score was only analysed as a continuous variable. 

### Potential confounders were clustered into three groups

#### Group 1: Family-related covariates

Family-related covariates included maternal and paternal educational level, household income, maternal and paternal BMI, whether the parents were divorced, and the number of siblings living with the child.

#### Group 2: Lifestyle covariates

Lifestyle covariates included total energy intake (kJ/day), total intake of fat (grams), total intake of carbohydrate (grams), and parental perception of the child’s physical activity level compared to peers.

#### Group 3: Family stress level

Analyses were adjusted for PSI score coded into three categories (“High”, “Medium”, “Low”), based on calculations of tertiles. 

Multiple linear regression analyses were performed in the statistical program SAS version 9.1, using the proc glm procedure. The analyses were done in four steps:

*Unadjusted model*, including the exposure, unadjusted for potential confounders.

*Model 1*, including exposure and all family-related covariates. 

*Model 2*, including exposure, all family-related covariates, and lifestyle covariates.

*Model 3*, including exposure, all family-related covariates, lifestyle covariates, and family stress level.

Since model 3 was conjectured to include all confounders on which information was available, this model was chosen as the final model. 

The residuals in each of the final models (model 3) were tested for normal distribution, using Kolmogorov-Smirnov tests. All final models were furthermore tested for linearity by plotting the residuals against the predicted values. 

A p-value of ≤ 0.05 was considered statistically significant. 

## Results

Baseline characteristics including PSB score, SDQ-TD score, age, BMI, BMI z-score, PSI score, maternal BMI, and paternal BMI in boys and girls, are shown in Table 1. T-tests showed that girls had a significantly higher PSB score than boys (Table 1). No other significant differences in baseline characteristics between boys and girls were found (Table 1).

**Table 1:** Baseline characteristics by gender.

	**Boys**					**Girls**					**p (t-test)**
	**n**	**Mean**	**SD**	**Min.**	**Max.**	**n**	**Mean**	**SD**	**Min.**	**Max.**	
**Age (years)**	360	4.0	1.1	2.1	6.3	275	4.0	1.1	2.1	6.4	0.41
**SDQ-TD score (points)**	329	6.8	4.0	0.0	21.0	253	6.5	3.8	0.0	27.0	0.37
**PSB score (points)**	330	7.4	1.9	0.0	10.0	253	8.2	1.6	4.0	10.0	< 0.001*
**BMI (kg/m^2^)**	360	16.4	1.3	12.9	23.0	275	16.3	1.4	12.2	23.7	0.28
**BMI z-score (SD)**	360	0.4	1.0	-3.1	4.5	275	0.4	1.0	-4.2	3.5	0.84
**PSI score (points)**	306	12.5	2.3	5.0	18.0	236	12.3	2.4	2.0	18.0	0.32
**Maternal BMI (kg/m^2^)**	323	26.3	5.5	17.0	45.0	245	27.2	5.5	17.1	44.6	0.07
**Paternal BMI (kg/m^2^)**	318	26.0	4.0	19.0	49.9	244	26.2	3.9	16.6	41.5	0.60

SDQ-TD score = Strengths and Difficulties Total Difficulties score PSB score = Prosocial Behaviour score

PSI score = Parental Stress Index score

In the unadjusted model, no association between SDQ-TD score and BMI z-score was found (Table 2). However, after adjusting for all potential confounders, there was a tendency towards a linear trend in the association between SDQ-TD score and BMI z-score (β = 0.021, p = 0.11) (model 3, Table 2). 

**Table 2:** SDQ-TD score, PSB score and body size.

	**BMI z-score (SD)**			
**SDQ-TD score**	**n**	**β**	**SD**	**p**
Unadjusted model	582	-0.001	0.01	0.93
Model 1	463	0.011	0.01	0.33
Model 2	432	0.014	0.01	0.25
Model 3	403	0.021	0.01	0.11
**PSB score**				
Unadjusted Model	583	-0.017	0.02	0.44
Model 1	464	-0.025	0.02	0.30
Model 2	433	-0.016	0.02	0.50
Model 3	404	-0.015	0.03	0.55

Model 1: Adjusted for maternal and paternal educational level, household income, maternal and paternal BMI, whether parents were divorced, and the number of siblings living with the child.

Model 2: Adjusted for maternal and paternal educational level, household income, maternal and paternal BMI, whether parents were divorced, the number of siblings living with the child, total energy intake, total intake of fat, total intake of carbohydrates, and physical activity.

Model 3: Adjusted for maternal and paternal educational level, household income, maternal and paternal BMI, whether parents were divorced, the number of siblings living with the child, total energy intake, total intake of fat, total intake of carbohydrates, physical activity, and family stress level.

SDQ-TD score = Strengths and Difficulties Total Difficulties score PSB score = Prosocial Behaviour score

No significant association was found between PSB score and BMI z-score, neither before nor after adjustments for potential confounders (Table 2). 

Compared to having a normal SDQ-TD score, neither a borderline nor an abnormal score was associated with BMI z-score in the unadjusted model (Table 3). However, compared to having a normal SDQ-TD score, an abnormal score was significantly associated with BMI z-score, after adjustments of all potential confounders (β = 0.36, p = 0.05) (model 3, Table 3). There was no interaction between SDQ-TD score and PSI score (p = 0.29, data not shown). 

**Table 3:** SDQ-TD score coded into 0-80^th^, 81^st^-90^th^, and 91^st^-100^th^ percentile and body size.

	**BMI z-score (SD)**			
	**n**	**β**	**SD**	**p**
**Unadjusted model**	582			
Normal score (0 - 80^th^ pct.)		0.00	-	-
Borderline score (81^st^-90^th^ pct.)		0.06	0.12	0.59
Abnormal score (91^st^-100^th^ pct.)		0.01	0.15	0.94
**Model 1**	463			
Normal score (0 - 80^th^ pct.)		0.00	-	-
Borderline score (81^st^-90^th^ pct.)		0.08	0.14	0.55
Abnormal score (91^st^-100^th^ pct.)		0.18	0.17	0.28
**Model 2**	432			
Normal score (0 - 80^th^ pct.)		0.00	-	-
Borderline score (81^st^-90^th^ pct.)		0.09	0.14	0.55
Abnormal score (91^st^-100^th^ pct.)		0.26	0.17	0.12
**Model 3**	403			
Normal score (0 - 80^th^ pct.)		0.00	-	-
Borderline score (81^st^-90^th^ pct.)		0.12	0.14	0.41
Abnormal score (91^st^-100^th^ pct.)		0.36	0.18	0.05*

Model 1: Adjusted for maternal and paternal educational level, household income, maternal and paternal BMI, whether parents were divorced, and the number of siblings living with the child.

Model 2: Adjusted for maternal and paternal educational level, household income, maternal and paternal BMI, whether parents were divorced, the number of siblings living with the child, total energy intake, total intake of fat, total intake of carbohydrates, and physical activity.

Model 3: Adjusted for maternal and paternal educational level, household income, maternal and paternal BMI, whether parents were divorced, the number of siblings living with the child, total energy intake, total intake of fat, total intake of carbohydrates, physical activity, and family stress level.

SDQ-TD score = Strengths and Difficulties Total Difficulties score PSB score = Prosocial Behaviour score

## Discussion

Our results suggested a direct association between SDQ-TD score and BMI z-score among young children. However, the results also suggested an increased body size particularly among those with most extreme SDQ-TD scores. This association, that was only present at high SDQ-TD scores, may explain why the linear association between SDQ-TD and BMI z-score shown in table 2 did not reach statistical significance. 

Previous cross-sectional studies have shown associations between SDQ-TD score or subscale scores and body size or obesity [19–22], which is in line with our results, but one study did not find an association [23]. To the best of our knowledge, prospective associations of SDQ with body size or obesity have not been published hitherto, and as cross-sectional studies do not allow conclusions regarding causality, longitudinal or experimental studies are needed. 

Previous research has provided evidence that psychological stress may be a determinant of overweight and obesity (and vice versa) [4,24]. This raises the question of whether the observed association between behavioural problems and BMI reflects that SDQ-TD scores, to some extent, reflect the child’s stress level. Indeed, children who are exposed to multiple risks are more likely to demonstrate behavioural problems than children who are exposed to single risks (e.g. risks potentiate one another) [25], as also reported in a review from 2010 (although the generalizability of the findings was limited) [24]. A fundamental part of the body’s neuroendocrine response to stress is mediated via the hypothalamus-pituitary-adrenal (HPA) axis [26]. The end product of the HPA axis is cortisol which is often used to examine the activity of the HPA axis [26,27]. Previous studies have found associations between behavioural problems and cortisol [28–32], suggesting that behavioural problems may reflect stress. Nonetheless, only one of these studies used SDQ to measure behavioural problems [31], and the extent to which SDQ-TD scores reflect stress is an open question. 

At a glance, the observed estimates may be considered weak. Yet, the mean SDQ-TD score for both boys and girls was approximately 7 points (Table 1). The estimated effects shown in Table 2 are per 1 point increase, but since the scores ranged from 0 to 21 in boys and 0 to 27 in girls there was a large variation in the SDQ-TD score, suggesting that some subgroups of children with a high SDQ-TD score may be at a particular high risk of becoming overweight or obese. 

Compared to previous studies, our study adds to the present knowledge in three ways: First, we examined the direct association between behavioural problems and body size, rather than behavioural factors correlating with both behaviour problems and obesity, such as food intake and physical activity. This may be important in relation to the search for determinants of overweight and obesity which are unrelated to energy balance. Second, the study was conducted among pre-school children. As described in the introduction, most previous studies published hitherto were conducted in school-aged children [4], and knowledge on the effect of behavioural problems in relation to overweight and obesity in young children has consequently hitherto been lacking. Knowledge of the age at which the association between behaviour and weight becomes significant may be central for optimal planning of preventative efforts including stress reduction. Finally, as far as we know this is the first study to examine associations between measurements of behavioural problems in children and body size in a group exclusively comprising children predisposed to overweight and obesity. The observation that behavioural problems are more common in children from low SES families, that are also predisposed to overweight and obesity [4], calls for further research into whether the association between stress and weight is restricted to low SES children, or is also present among other groups of children predisposed to overweight and obesity, for example in children with overweight or obese 1^st^ degree relatives. 

Behavioural problems (and the different dimensions of behavioural problems) may be caused by genetic or environmental factors, or gene-environment interactions. Whereas approximately half of the population variance in aggressive behaviour can be explained by genetic processes, environment has been found to explain more of the variance in prosocial behaviour than heritability [25]. Stressful life events, such as poverty, problematic parenting, parental divorce, or marital conflicts are examples of environmental factors which could lead to non-specific behavioural problems in children [25,33]. Specific types of environmental adversities are not associated with one specific type of reaction, since children’s reactions to stress are substantially heterogeneous [25]. This heterogeneity may reflect genetic and non-genetic vulnerability in the child, or reflect complex interactions among different factors in the child’s environment [25]. As a consequence of this heterogeneity, a low SDQ-TD score does not reflect that the child will not have been exposed to environmental adversities, but may be an indicator of resilience (e.g. the child is doing well despite being exposed to substantial environmental stress). However, this may not be important in the present study, as our research question addresses actual behavioural problems irrespective of the causal factors influencing the child’s behaviour. 

The evaluation of behavioural problems in children in relation to child body size, and the possibility to adjust the analyses for a large number of potential confounders related to lifestyle and socio-demographics are some of the methodological strengths of the present study. 

Our study also has some limitations. For example, information on which parent (mother or father) completed the questionnaire was not obtained. Although it has been suggested that perceived stress does not differ between mothers and fathers [34], other studies found higher levels of parental stress in mothers compared to fathers [35–37]. If we assume that mothers were more likely to complete the questionnaire, this may have introduced information bias, which may have led to attenuation of our results. Since predisposition to overweight and obesity can be multi-causal, ranging from biological risk factors such as high birth weight or genetics, to social risk factors such as ethnicity or socioeconomic position [38], child behavioural problems may not be a determinant of overweight and obesity in all predisposed groups. Our results may therefore not be generalizable to all predisposed individuals per se. The participation rate of 20% may have led to differential selection, consequently curtailing the external validity of our results. It may be argued that families of children with behavioral problems could be less likely to participate in the study, which however would have led to attenuation, rather than inflation, of our results. 

Finally, reporting bias in relation to SDQ score is a possibility. Parents may, intentionally or unintentionally, underreport their child’s behavioural problems when completing the questionnaires. Since problem behaviours may be highly situational, the rater’s perception of a given situation may influence the ratings, and one type of problem behaviour in the child may influence the perception and rating of other behaviours (“halo-effect”) [15,39]. If reporting bias has influenced our data, the results may have been attenuated rather than inflated. 

## Conclusion

Our results suggest that SDQ-TD score and BMI z-score are related, in particular for those children with the highest levels of behavioural problems. There were no significant associations between PSB score and BMI z-score. 

## Supporting Information

Table S1 **Questions selected and modified from the Swedish version of the Parental Stress Index (translated into English from Danish).**
(DOCX)

Appendix S1 **Questions selected and modified from the Swedish version of the Parental Stress Index (translated into English from Danish).**
(PDF)
