# Supplementary material for: Child Behavioural Problems and Body Size among 2-6 Year Old Children Predisposed to Overweight. Results From the “Healthy Start” Study
Source: PLoS One. 2013 Nov 8;8(11):e78974. doi: 10.1371/journal.pone.0078974 (PMC3826721; doi:10.1371/journal.pone.0078974)
Supplement: Table S1 — Questions selected and modified from the Swedish version of the Parental Stress Index (translated into English from Danish). (DOCX) [file pone.0078974.s001.docx]

**Table S1:** Questions selected and modified from the Swedish version of the Parental Stress Index (translated into English from Danish)

Which changes have been in your life since you had children? (Put one mark in each category)

□ Less sleep

□ More sleep

□ No change

□ Less work

□ More work

□ No change

□ Less stress

□ More stress

□ No change

□ Fewer social gatherings in the home

□ More social gatherings in the home

□ No change

□ Fewer worries

□ More worries

□ No change

□ Less joy of life

□ More joy of life

□ No change

□ Less time to yourself

□ More time to yourself

□ No change

□ Less everyday surplus energy

□ More everyday surplus energy

□ No change

□ Fewer household conflicts

□ More household conflicts

□ No change

□ Less complexity of being a parent compared to expectations

□ More complexity of being a parent compared to expectations

□ As expected

Additional information:

________________________________________________________________________________________________________________________________________________________________________________________________________________________________________________
